# Worrying in the wings? Negative emotional birth memories in mothers and fathers show similar associations with perinatal mood disturbance and delivery mode

**DOI:** 10.1007/s00737-019-00973-5

**Published:** 2019-07-06

**Authors:** Claire Hughes, Sarah Foley, Rory T. Devine, Andrew Ribner, Lara Kyriakou, Lucy Boddington, Emily A. Holmes, NewFAMS team Creators/Copyright Holders, Claire Hughes, Rory T. Devine, Judi Mesman, Clancy Blair, NewFAMS team Contributors, Lenneke Alink, Marjolein Branger, Wendy Browne, Rosanneke Emmen, Sarah Foley, Lara Kyriakou, Anja Lindberg, Gabrielle McHarg, Andrew Ribner, Mi-Lan Woudstra

**Affiliations:** 1 Centre for Family Research, University of Cambridge, Cambridge, UK; 2 School of Psychology, University of Birmingham, Birmingham, UK; 3 Department of Applied Psychology, NYU Steinhardt, New York University, New York, NY USA; 4 Division of Psychology, Department of Clinical Neuroscience, Karolinksa Institutet, Solna, Sweden

**Keywords:** Birth experience, Depression, Anxiety, Mothers, Fathers, Delivery

## Abstract

Negative birth experiences can lead to symptoms of post-traumatic stress disorder in new mothers but have received much less attention in new fathers. A sample of 314 first-time expectant couples rated their symptoms of anxiety and depression in the third trimester and at 4-month post birth (227 vaginal delivery, 87 caesarean section), when they also completed the emotional memories subscale of the BirthMARQ (Foley et al. BMC Pregnancy Childbirth, 14, 211, 2014). We first examined mode of delivery (vaginal birth versus caesarean section) as a predictor of mothers’ and fathers’ BirthMARQ scores. Next, we used actor-partner interdependence model (APIM) to investigate intra- and interpersonal associations between birth experiences and maternal/paternal latent factors for antenatal and postnatal depression/anxiety. Reports of negative birth experiences were more common for mothers than fathers and for parents of babies born by caesarean section than by vaginal delivery. Within-couple agreement was moderately strong and, for both parents at both time-points, individual differences in negative birth memories were associated with symptoms of depression and anxiety. Negative birth memories also played a mediating role in the association between birth via caesarean section and reduced postnatal maternal wellbeing. Given the striking similarities between mothers and fathers in links between birth experiences and wellbeing, our findings highlight the need for partner-inclusive intervention strategies.

**Electronic supplementary material:**

The online version of this article (10.1007/s00737-019-00973-5) contains supplementary material, which is available to authorized users.

While often eagerly anticipated, the transition to parenthood can be very stressful. Indeed, numerous studies have reported significant declines in well-being for both mothers and fathers (Paulson and Bazemore 2010; Philpott et al. 2017). At its most extreme, perinatal mood disturbance is, via suicide, a leading cause of maternal death (Knight and Tuffnell 2018). Less severe symptoms adversely affect parenting quality (Tronick and Reck 2009) and predict problems in infant cognitive development (Fredriksen et al. 2018; Murray et al. 1996) and child adjustment (for a meta-analytic review, see Goodman et al. 2011). Indeed, more than one in four women with difficult birth experiences report symptoms of post-traumatic stress disorder (Czarnocka and Slade 2000); these include intrusive thoughts or images, avoidance of situations associated with birth (including avoiding the baby), blunted affect and low mood (Dikmen-Yildiz et al. 2018). This study therefore aimed to elucidate the relations between wellbeing and negative birth memories in first-time parents.

Demographic shifts add to the urgency of this research challenge. Specifically, increases in mean maternal age and body mass index are likely to increase the prevalence of traumatic birth (i.e. one in which there is a significant threat to life or injury to mother or child, such as an emergency caesarean section) (Kim et al. 2018). However, as Bell and Andersson (2016) noted in their systematic review, several methodological weaknesses limit research into associations between birth experiences and postnatal wellbeing. These include small samples, failure to control for factors related to postnatal depression (e.g. anxiety) or to include either antenatal ratings of wellbeing or validated instruments to assess birth experiences. Addressing the last of these concerns, Foley et al. (2014) have developed the Birth Memories and Recall Questionnaire (BirthMARQ) and assessed its validity in an online study of 523 women contacted in the first year after childbirth. Consistent with previous findings (e.g. Watson et al. 2012) and supporting the BirthMARQ’s validity as a screen for emotion dysregulation following difficult birth experiences, women who scored above cut-offs on the Edinburgh Postnatal Depression Scale (Cox et al. 1987) reported more negative emotional memory, greater centrality of memories and involuntary recall (Foley et al. 2014).

As noted by Foley et al. (2014), an important but under-researched topic concerns the impact of fathers’ experiences as birth partners on paternal wellbeing. In a meta-synthesis of 120 fathers’ multi-dimensional experiences as birth partners, Johansson et al. (2015) reported that fathers are commonly left ‘in the wings’ feeling overwhelmed or inadequate. Yet despite growing evidence that exposure to paternal symptoms of depression has a negative impact on infant development and adjustment (Ramchandani et al. 2008), very few studies have examined the affective correlates of fathers’ negative birth experiences. In a notable exception, Gürber et al. (2017) followed 189 Swiss heterosexual couples from the last trimester of pregnancy to 1- and 4-week post-partum. For both mothers and fathers, negative birth experiences and antenatal depressive symptoms were each related to poor psychological postpartum adjustment (i.e. postnatal depression and birth-related trauma). Moreover, self-reported birth trauma and postnatal (but not antenatal) symptoms of depression showed a moderate within-couple association. However, compared with fathers, mothers reported higher levels of the following: (a) birth trauma at both follow-up visits and (b) depressive symptoms in the third trimester and at the second (4-week) follow-up visit. In addition, although fathers’ birth experiences were related to mothers’ symptoms of birth-related trauma, there was no association between mothers’ birth experiences and fathers’ symptoms of birth-related trauma.

The current study builds on the above work in three ways. First, given the well-established increase in anxiety across the transition to parenthood (Matthey et al. 2003), we included symptoms of anxiety as well as depression in the antenatal and postnatal wellbeing measures in order to achieve a more complete picture of associations between birth experiences and wellbeing. Second, we compared BirthMARQ ratings for couples who differed in mode of birth (vaginal delivery versus caesarean section). Third, we applied Actor Partner Interdependence Modelling (Cook and Kenny 2005) to examine the influence of partner experience on parent wellbeing.

In sum, our first aim was to compare BirthMARQ responses across parent gender and mode of birth (vaginal delivery/caesarean section). We predicted that negative emotional memories of birth would be more likely for the following: (a) mothers compared with fathers and (b) caesarean section compared with vaginal delivery. Our second aim was to examine actor and partner effects on relations between parents’ wellbeing, mode of delivery and birth memories.

## Method

### Participants

Participants were recruited from two sites (i.e. the UK and USA) between November 2014 and October 2015 as part of a larger multi-site study examining the links between parental wellbeing, parent-child interactions and child outcomes. To be eligible participants, they had to be as follows: (1) first-time parents, (2) expecting a healthy singleton baby, (3) planning to speak English as a primary language with their child and (4) without any history of severe mental illness or substance misuse. In total, 343 couples expecting their first child were recruited via ultrasound clinics, birthing classes and hospital visits in the East of England and in New York City. Eight of the 213 British couples recruited were ineligible at 4 months, due to serious birth complications or moving long distance. Of the remaining sample, 196 (96%) families agreed to a postnatal home visit (109 boys, 87 girls; *M*_Age_ = 4.12 months, SD = 0.39 months, range 2.97–5.63 months). In the USA, 130 couples were recruited, of whom one family had moved out of the area at 4 months; 126 (97%) of the remaining 129 families agreed to a home visit (57 boys, 69 girls, *M*_Age_ = 4.60 months, SD = 0.52 months, range 3.53–7.50 months), though questionnaire data was only available for 123 families. To streamline analyses, data from these two sites were pooled, with country included as a dummy variable. For more detailed participant information, see Supplementary Material 1.

In our sample of 319 families, mothers were, on average, 33.04 years old, SD = 3.64, range 25.40–43.68 years, at the birth of their baby. Fathers were, on average, 34.59 years old, SD = 4.56, range 24.04–51.87 years. The sample was predominantly highly educated (85% of mothers and 75.5% of fathers had an undergraduate or higher degree). A minority of parents were from ethnic minority backgrounds (21.3% of mothers and 18.2% of fathers). Most mothers gave birth vaginally (*n* = 227), but just over quarter (*n* = 87) gave birth via caesarean section (*n* = 71 emergency and *n* = 16 elective, note birth records were incomplete for five women).

### Procedure

The National Health Service (NHS UK) Research Ethics Committee (name blinded) and the University Committee on Activities Involving Human Subjects at (university name blinded) approved the study protocol (ref number blinded). Parents provided informed consent to take part in in-person interviews in the third trimester and at 4-month post-birth and also provided demographic information and completed wellbeing questionnaires online.

### Measures

#### Birth experiences

Mothers and fathers completed the five-item Emotional Memory section of the Birth Memories and Recall Questionnaire (BirthMARQ) questionnaire (Foley et al. 2014), which focused on parents’ positive, negative or mixed emotions at the birth/in recalling the event. Each item (e.g. ‘my emotions at the time were extremely negative’) was rated on a scale ranging from 1 (strongly disagree) to 7 (strongly agree). With the two positive emotion items reverse-scored, the five items were summed to create a ‘negative birth experiences’ subscale, which had a possible range of 5 to 35 and showed good internal consistency in both mothers, *α* = .87 and fathers, *α* = .86.

#### Parental wellbeing

Mothers and fathers reported depression and anxiety symptoms using the 12-item General Health Questionnaire (GHQ; Goldberg et al. 1997), the 20-item Centre for Epidemiological Studies Depression Scale (CESD; Radloff 1977) and the 6-item State-Scale of the State-Trait Anxiety Inventory (STAI; Spielberger et al. 1983). High scores indicated problems for each measure that, as shown in Table 1, all showed good internal consistency at both time-points.

**Table 1:** Descriptive statistics and reliability information for maternal and paternal questionnaires from time 1 to time 2

		Mother								Father							
		Third trimester				4-month postnatal				Third trimester				4-month postnatal			
		*M*	SD	*N*	*α*	*M*	SD	*N*	*α*	*M*	SD	*N*	*α*	*M*	SD	*N*	*α*
UK	CESD	9.76	5.84	195	.81	8.75	6.93	189	.87	7.83	6.16	192	.84	9.12	6.91	178	.87
	GHQ	1.92	2.10	195	.76	1.57	2.19	190	.81	1.49	2.11	192	.80	2.26	1.74	182	.81
	STAI	10.70	2.93	195	.77	10.21	2.88	189	.77	11.16	2.73	192	.72	11.15	3.13	179	.81
	BirthMARQ	–	–	–	–	17.15	7.26	188	.86	–	–	–	–	14.30	7.09	176	.86
USA	CESD	9.79	5.40	121	.76	9.22	7.19	116	.86	9.13	6.51	115	.84	11.12	8.96	103	.90
	GHQ	1.84	2.01	119	.72	2.07	2.30	109	.77	1.55	2.25	115	.82	2.62	3.21	99	.89
	STAI	10.92	3.02	122	.78	11.90	3.09	114	.77	11.87	2.99	115	.75	12.01	3.82	103	.85
	BirthMARQ	–	–	–	–	14.56	7.06	114	.85	–	–	–	–	10.70	5.71	101	.85

*CESD*, Center for Epidemiological Studies Depression Scale; *GHQ*, General Health Questionnaire; *STAI*, State-Trait Anxiety Inventory; *Birth MARQ*, Birth Memories and Recall Questionnaire

## Results

### Comparing BirthMARQ scores across parent gender and mode of delivery

**Fig. 1:**
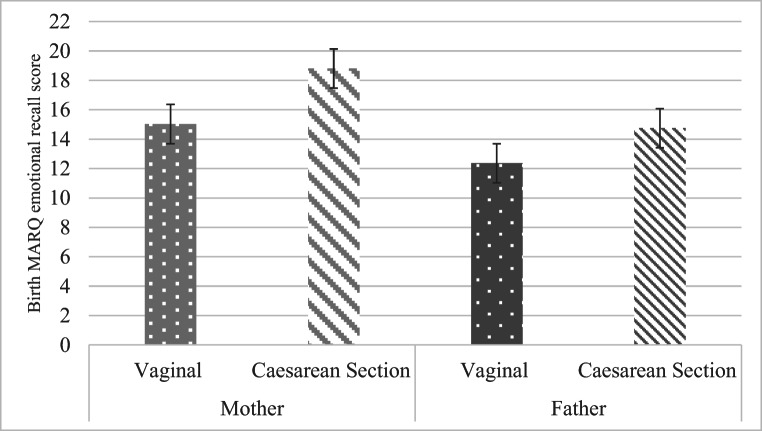
Mean BirthMARQ scores are shown by mode of birth (V, vaginal delivery versus C, caesarean section) for mothers and fathers

Table 1 presents descriptive statistics for all study questionnaire measures. Mean BirthMARQ scores are shown by mode of birth (V, vaginal delivery versus C, caesarean section) for mothers and fathers in Fig. 1. Across mothers and fathers, the mean correlation between Birth MARQ scores and age was − .03, range = − .07 to .07, *p > .*05 for all. Likewise, separate *t* tests showed no education-related contrast in BirthMARQ scores for mothers, *t*(287) = .53, *p* = .599, Cohen’s *d* = 0.09, or fathers, *t*(277) = .27, *p* = .784, Cohen’s *d* = 0.10.

### Intra- and inter-personal effects in links between BirthMARQ scores and wellbeing

Given strong within-person correlations (see Table 2), confirmatory factor analysis (CFA) in M*plus* 7 (Muthén and Muthén 2017) was performed on the raw scores of each indicator to create a single latent factor for depressive and anxiety symptoms for mothers and fathers at each time-point. Note that the lead indicator intercept was set to 0 for each latent factor so that each latent factor mean could be freely estimated (Geiser 2013). A single measurement model of correlated latent factors for mothers and fathers across the two time points provided a good fit, *χ*^2^ (214) = 341.161, *p* < .001, RMSEA = 0.038, CFI = 0.969, TLI = 0.960.

**Table 2:** Correlations between measures (maternal below diagonal and paternal above diagonal) from time 1 to time 2

	T2						
	T1 CESD	T1 GHQ	T1 STAI	T2 CESD	T2 GHQ	T2 STAI	Birth MARQ
T1 CESD	–	.70**	.58**	.56**	.37**	.42**	.14*
T1 GHQ	.60**	–	.46**	.38**	.43**	.28**	.08
T1 STAI	.49**	.39**	–	.36**	.28**	.44**	.14*
T2 CESD	.48**	.30**	.22**	–	.70**	.70**	.18**
T2 GHQ	.23**	.24**	.10	.61**	–	.57**	.15*
T2 STAI	.29**	.21**	.26**	.56**	.51**	–	.19**
Birth MARQ	.12*	.16**	.09	.18**	.12*	.07	–

*T1*, third trimester pregnancy; *T2*, 4-month postnatal; *CESD*, Centre for Epidemiological Studies Depression Scale; *GHQ*, General Health Questionnaire; *STAI*, State-Trait Anxiety Inventory; *Birth MARQ*, Birth Memories and Recall Questionnaire (completed at T2)

*p < .05, **p < .01

Next, we used an actor-partner interdependence model (APIM, Cook and Kenny 2005) to examine associations between couples and links between antenatal wellbeing, mode of delivery, birth experience and postnatal wellbeing. An APIM accounts for the inherently related nature of data from couples (Cook and Kenny 2005) and allows exploration of actor effects (i.e. intrapersonal) and partner effects (i.e. interpersonal) on outcomes. Following this, we examined whether birth experience mediated the association between mode of delivery and poor wellbeing at 4 months. A model to test for this indirect effect was specified in M*plus* using bootstrapping procedures (5000 bootstrap samples) (Hayes 2009). Model parameters and standard errors were estimated in M*plus* using all available data (Muthén and Muthén 2017). Please see Supplementary Material 2 for detail of model construction and testing.

Our unconstrained APIM examining intra- and inter-personal effects of antenatal wellbeing and birth experience on postnatal wellbeing showed a good fit to the data, RMSEA = 0.05, CFI = 0.93 and TLI = 0.91, and explained 21.5% of the variance in mothers’ postnatal wellbeing and 31.1% of the variance in fathers’ postnatal wellbeing. To test for gender differences in the strength of the pathways, model constraints were built up so that in turn, all pathways were constrained to equality and changes to model fit were examined. Compared to the baseline model, a nested model constraining all pathways to equality did not provide a better fit to the data, Δ*χ*^2^ (6) = 35.54, *p* < .000, suggesting the strength of the associations between antenatal wellbeing and birth experience and postnatal wellbeing was different for mothers and fathers. A model freeing the autoregressive pathways (i.e. postnatal wellbeing on antenatal wellbeing), while constraining all other pathways to equality did not significantly worsen model fit when compared to the baseline model, Δ*χ*^2^ (3) = 2.86, *p* = .414, suggesting that there was greater stability in wellbeing across the transition to parenthood for fathers than for mothers.

**Fig. 2:**
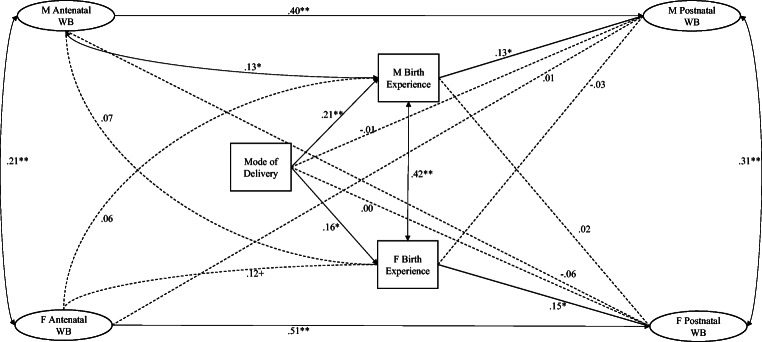
Actor-partner interdependence model of pathways between parental wellbeing, mode of delivery and birth experience. Standardised estimates displayed. M, mother; F, father; WB, wellbeing latent factor. Black lines represent significant pathways and dashed lines represent non-significant pathways. +*p* < .10, *p < .05, **p < .01

Our unconstrained APIM (Cook and Kenny 2005) is illustrated by the standardised path coefficients in Fig. 2. Overall, this model highlights five findings. First, across the transition to parenthood individual differences in wellbeing were moderately stable for mothers, *β* = 0.40, 95%CI [.23, .54], *p* < .000, and highly stable for fathers, *β* = 0.51, 95%CI [.36, .64], *p* = .000. Second, in comparison with the modest within-couple associations in self-reported wellbeing at antenatal, *β* = 0.21, 95%CI [.07, .34], *p* = .012 and postnatal visits, *β* = 0.31, 95%CI [.16, .44], *p* < .000, the moderately strong within-couple association in BirthMARQ scores, *β* = 0.42, 95%CI [.33, .51], *p* < .000, indicates good agreement between parents. Third, there was an effect of country on mothers’ wellbeing at 4 months, *β* = − 0.15, 95%CI [− .25, − .04], *p* = .022, but no effect of race on either mothers’ or fathers’ wellbeing at 4 months. Fourth, the actor pathways between poor antenatal wellbeing and negative birth experiences appeared stronger (though not significantly different in strength) for mothers, *β* = 0.13 95%CI [.03, .24], *p* = .036, than fathers, *β* = 0.12 95%CI [− .05, .18], *p* = .081. Actor pathways between negative birth memories and poor wellbeing at 4 months were also similar in strength for mothers, *β* = 0.13 95%CI [.03, .24], *p* = .035, and fathers, *β* = 0.15 95%CI [.06, .26], *p* = .009. Finally, the total indirect effect of mode of birth via negative birth experience on postnatal wellbeing was significant, *β* = .73 95%CI [.25, 1.15], *p* = .026, and evident for both mothers, *β* = .37 95%CI [.10, .80], and fathers, *β* = .37 95%CI [.09, .90]. Additional analyses excluding mothers with elective rather than emergency C-sections yielded very similar results.

## Discussion

In this study of 319 first-time parents, BirthMARQ ratings of negative emotional memories of birth gathered at 4 months were as follows: (a) compared across parent gender and mode of birth delivery and (b) examined in relation to symptoms of anxiety and depression both in the third trimester of pregnancy and at 4-month post-birth. As expected, parents of babies born by caesarean section reported higher BirthMARQ scores than parents of babies born by vaginal delivery, as did mothers, compared with fathers. Within-couple agreement was moderately strong for BirthMARQ scores, which were significantly related to individual differences in wellbeing at both time-points for both parents, even when the stability of individual differences in wellbeing was taken into account. Below, we discuss each of these findings in turn.

Adding rigour to a relatively novel research topic, the latent variables for symptoms of anxiety/depression demonstrated that negative memories of birth showed strikingly similar associations with wellbeing for both mothers and fathers. For example, BirthMARQ scores were associated with postnatal wellbeing for both mothers and fathers even when the stability of individual differences in wellbeing was taken into account. This finding highlights the need to extend research on the psychological consequences of difficult birth experiences to include fathers. Antenatal distress was also significantly associated with later reports of negative birth memories for mothers and marginally associated fathers. Arguably then, the epithet ‘worrying in the wings’ applies both to new mothers and to new fathers. Importantly, the association between antenatal wellbeing and BirthMARQ scores in this study was similar in magnitude to the contrast between vaginal delivery and caesarean section. Note also that even within this well-educated, relatively affluent sample of first-time heterosexual cohabiting parents, there was substantial variation in antenatal wellbeing. That said, it is worth noting that the relatively small size of the New York sub-sample precluded any statistical tests of the conceptual equivalence of BirthMARQ scores across sites; addressing this issue in larger studies is therefore a valuable direction for future research.

As shown in a recent study (Favrod et al. 2018), spontaneous negative imagery of impending birth is associated with greater fear of childbirth, indicating that expectant parents’ thoughts can colour their emotional experiences related to giving birth. Recent meta-analytic findings also demonstrate that expectant parents’ thoughts and feelings about their unborn child (assessed via interview or questionnaire) predict maternal sensitivity in the first year of life (Foley and Hughes 2018). An interesting direction for future research would be to assess whether difficult birth experiences amplify or attenuate this association between antenatal thoughts and postnatal sensitivity.

## Conclusions

Three key conclusions emerged from this study of 638 new parents living in the UK and in the USA. First, mothers and fathers showed strikingly similar links between negative emotional memories of birth and poor antenatal wellbeing, suggesting that healthcare practitioners should adopt a partner-inclusive approach within antenatal and postnatal care. For example, building on recent review evidence from de Graaff et al. (2018) that highlights the efficacy of writing interventions to reduce symptoms of birth-related PTSD, our findings not only demonstrate that the BirthMARQ shows real promise as a tool for identifying new parents who might benefit from extra support, but also suggest that these interventions should include new fathers and new mothers.

Second, the similarity in magnitude between antenatal wellbeing and mode of birth delivery (vaginal or caesarean section) as predictors of parents’ BirthMARQ scores deserves note. Psychological wellbeing is not yet routinely assessed within antenatal care (and is completely lacking with regard to fathers). Our finding therefore highlights the importance of attending to the wellbeing of both expectant mothers and fathers and, where possible, implementing interventions that foster wellbeing prior to the transition to parenthood.

Third, the mediation effect of negative birth experiences in the association between mode of delivery and postnatal wellbeing highlights the need to take steps to reduce the trauma associated with giving birth by caesarean section (e.g. helping parents understand the diversity of birth experiences such that they do not set themselves unrealistic goals with regard to birth). Crucially, these conversations should include both parents and may also enable fathers to provide a supportive presence at delivery.

### Electronic supplementary material

ESM 1 (DOCX 13 kb)

ESM 2 (DOCX 13 kb)

## Appendix

NewFAMS team Creators/Copyright Holders:

Claire Hughes, University of Cambridge, UK.

Rory T. Devine, University of Birmingham, UK.

Judi Mesman, Leiden University, Netherlands.

Clancy Blair, New York University, USA.

NewFAMS team Contributors:

Lenneke Alink, Leiden University, Netherlands.

Marjolein Branger, Leiden University, Netherlands.

Wendy Browne, University of Cambridge, UK.

Rosanneke Emmen, Leiden University, Netherlands.

Sarah Foley, University of Cambridge, UK.

Lara Kyriakou, New York University, USA.

Anja Lindberg, University of Cambridge, UK.

Gabrielle McHarg, University of Cambridge, UK.

Andrew Ribner, New York University, USA.

Mi-Lan Woudstra, Leiden University, Netherlands.
