# Supplementary material for: Worrying in the wings? Negative emotional birth memories in mothers and fathers show similar associations with perinatal mood disturbance and delivery mode
Source: Arch Womens Ment Health. 2019 Jul 6;23(3):371–7. doi: 10.1007/s00737-019-00973-5 (PMC7244466; doi:10.1007/s00737-019-00973-5)
Supplement: Supplementary file 1 — (DOCX 13 kb) [file 737_2019_973_MOESM1_ESM.docx]

**Supplementary Material 1**

**Participants**

Participants were recruited from two sites (i.e., UK and USA) between November 2014 and October 2015 as part of a larger multi-site study examining the links between parental wellbeing, parent-child interactions and child outcomes. To be eligible participants had to be: (1) first-time parents, (2) expecting a healthy singleton baby, (3) planning to speak English as a primary language with their child and (4) without any history of severe mental illness or substance misuse. In total, 343 couples expecting their first child were recruited via ultrasound clinics, birthing classes, and hospital visits in the East of England and in New York City. Eight of the 213 British couples recruited were ineligible at 4 months, due to serious birth complications or moving long-distance. Of the remaining sample, 196 (96%) families agreed to a postnatal home visit (109 boys, 87 girls; *M*_Age_ = 4.12 months, *SD* = 0.39 months, range: 2.97 – 5.63 months). In the US, 130 couples were recruited, of whom one family had moved out of the area at 4 months; 126 (97%) of the remaining 129 families agreed to a home visit (57 boys, 69 girls, *M*_Age_ = 4.60 months, *SD* = 0.52 months, range: 3.53 – 7.50 months), though questionnaire data was only available for 123 families.

The participants in these two sites differed significantly in terms of age, education and mode of birth delivery. Specifically, UK mothers were, on average 32.61 years old at the birth of their baby, *SD* = 3.60, range: 25.10 – 43.15 years. In contrast, New York mothers were, on average 34.18 years old, *SD* = 3.93, range: 26.64 – 55.01 years; *t*(301) = 3.35,  *p* <.01, Cohen’s *d =* 0.40. Likewise, UK fathers were, on average, 33.98 years old, *SD* = 4.35, range: 24.05 – 49.63 years as compared with US fathers who were, on average 35.86 years old, *SD* = 4.85, range: 28.96 – 55.95 years; *t*(287) = 3.64,  *p* <.01, Cohen’s *d =* 0.45. While parents in both sites were highly educated, the proportion of parents with an undergraduate or higher degree was significantly higher in the USA for both mothers (97.5% for USA mothers *versus* 84.6% of UK mothers; χ^2^ = 11.29, *p* < .01) and fathers (94.8% of USA fathers *versus* 77.1% of UK fathers; χ^2^ = 16.62, *p* < .001). In terms of ethnic background, 9% of UK mothers and 26% of USA mothers were from ethnic minorities; χ^2^ = 19.83, *p* < .001; corresponding percentages for fathers in the UK and USA were 7% and 19%; χ^2^ = 13.12, *p* < .001. Finally, significantly more babies were born by caesarean section in the USA (37%) than in the UK (21%; χ^2^ = 12.0, *p* <.01).
