# Supplementary material for: Worrying in the wings? Negative emotional birth memories in mothers and fathers show similar associations with perinatal mood disturbance and delivery mode
Source: Arch Womens Ment Health. 2019 Jul 6;23(3):371–7. doi: 10.1007/s00737-019-00973-5 (PMC7244466; doi:10.1007/s00737-019-00973-5)
Supplement: Supplementary file 2 — (DOCX 13 kb) [file 737_2019_973_MOESM2_ESM.docx]

**Supplementary Material 2**

Model Construction and Testing

We used an Actor-Partner Interdependence Model (APIM: Cook & Kenny, 2005) to examine associations between couples and links between antenatal wellbeing, mode of delivery, birth experience and postnatal wellbeing. We regressed the latent factor for maternal and paternal postnatal wellbeing on the latent factor for both maternal and paternal antenatal wellbeing and birth experience. We also regressed latent factor for postnatal wellbeing on mode of delivery and also included country and race as dummy variables. Following this, we examined whether birth experience mediated the association between mode of delivery and poor wellbeing at 4 months. A model to test for this indirect effect was specified in M*plus* using bootstrapping procedures (5,000 bootstrap samples) (Hayes, 2009). Model fit was tested using Brown’s (2006) recommended criteria: root mean square error of approximation (RMSEA) ≤ 0.06, comparative fit index (CFI) ≥ 0.90 and Tucker-Lewis Index (TLI) ≥ 0.90. As we used a robust maximum likelihood estimator, we used the Satorra-Bentler $\chi$^2^ difference test (Satorra & Bentler, 2010) to calculate the difference between each nested model and the comparison model. We used a full information approach under the assumption that data were missing at random so that all eligible families who participated prenatally and had partial data for the postnatal follow-up phase (*N* = 314) were included. Model parameters and standard errors were estimated in M*plus* using all available data.
